# Predicting and Weighting the Factors Affecting Workers’ Hearing Loss Based on Audiometric Data Using C5 Algorithm

**DOI:** 10.5334/aogh.2522

**Published:** 2019-06-18

**Authors:** Sajad Zare, Mohammad Reza Ghotbi-Ravandi, Hossein ElahiShirvan, Mostafa Ghazizadeh Ahsaee, Mina Rostami

**Affiliations:** 1 Department of Occupational Health, School of Public Health, Kerman University of Medical Sciences, Kerman, IR; 2 Student Research Committee, Kerman University of Medical Sciences, Kerman, IR; 3 Department of Computer Engineering, Shahid Bahonar University of Kerman, IR

## Abstract

**Introduction:**

With the extensively spread of industrialization in the world, noise exposure is becoming more prevalent in the industrial settings. The most important and definite harmful effects of sound include hearing loss, both permanent and temporary.

**Objective:**

This study was designed aimed to use the C5 algorithm to determine the weight of factors affecting the workers’ hearing loss based on the audiometric data.

**Methods:**

This cross-sectional, descriptive, analytical study was conducted in 2018 in a mining industry in southeastern Iran. In this study, workers were divided into three exposed groups with different sound pressure levels (one control group and two case groups). Audiometry was conducted for each group of 50 persons; hence, the total number of subjects was 150. The stages of this study include: 1) selecting factors (predictive) to check and weigh them; 2) conducting the audiometry for both ears; 3) calculating the permanent hearing loss in each ear and permanent hearing loss of both ears; 4) classifying the types of hearing loss; and 5) investigating and determining the weight of factors affecting the hearing loss and their classification based on the C5 algorithm and determining the error and accuracy rate of each model. To assess and determine the factors affecting the hearing loss of workers, the C5 algorithm and IBM SPSS Modeler 18.0 were used. SPSS V.18 was used to analyze the linear regression and paired t-test tests, too.

**Results:**

The results showed that in the first model (SPL <70 dBA), the 8KHz frequency with the weight of 31% had the highest effect, the factors of work experience and the frequency of 250Hz each with the weight of 3%, had the least effect, and the accuracy of the model was 100%. In the second model (SPL 70–80 dBA) the frequency of 8KHz with the weight of 21% had the highest effect, the frequency of 250Hz and the working experience each had the lowest effect with the weight of 7% and the accuracy of the model was calculated as 100%. In the third model (SPL >85 dBA), the 4KHz frequency with the weight of 31% had the highest effect, and the work experience with a weight of 1% had the lowest effect, and the accuracy of the model was 94%. In the fourth model, the 4KHz frequency with the weight of 22% had the highest effect and 250Hz and age each with the weight of 8% had the lowest effects; the accuracy of this model was calculated to be 99.05%.

**Conclusions:**

During investigating and determining the weight of the factors affecting hearing loss by the C5 algorithm, the high weight and effect of the 4KHz frequency were predicted in hearing loss changes. Considering the high accuracy obtained in this modeling, this algorithm is a suitable and powerful tool for predicting and modeling the hearing loss.

## Introduction

Significant evidence exists indicating that noise is among the most prevalent kinds of occupation-related dangers in today’s world. Research shows that, each day in Europe, about 450 million people experience noise levels of minimum 55 dB (A), 113 million individuals are exposed to a noise level of at least 64 dB (A), and 9.7 million persons experience the noise levels of 75 dB (A) or higher [1]. More than 30 million workers, in the United States, are exposed to hazardous noises and 7.4–10.2 million industrial workers are at risk of hearing loss resulted by occupational noise [2]. According to WHO standards, in Germany, around 4 to 5 million workers (constituting 12% to 14% of the country’s population) are exposed to excessive sound pressure levels. The majority of work-related activities are along with a proportion of noise; however, some of these activities are conducted through excessive sound pressure levels [3].

Excessive noise causes physiological complications such as hypertension, adrenaline production, an increased risk of heart attack, high blood pressure, changes in respiratory rate, and the amount of consumed oxygen, affecting the auditory system, and increased stomach and intestinal activities, and other social and economic effects, and influences the efficiency and effectiveness of the exposed people. It also leads to interferences with verbal communication and perceptions of warning signs and affects the safety and performance of individuals [4,5]. Although Cognitive function is affected by many environmental risk factors, but the environmental noise is more effective among these risk factors [6,7]. The mental effects of the noise include irritability, headache, fatigue, difficulty in concentration, feeling pressure in the head and eyelids, sleeping disorders and psychosis [8].

The extreme sound may lead to temporary or permanent hearing threshold change with the macroscopic (tympanic membrane rupture, acicular chain dislocation, perilymph fistula, etc.) and microscopic (tectorial and basilar membrane rupture, hair cell loss, etc.) effects in the ear [9]. Hearing damage may also result in the downstream variations in central auditory processing, such as the variations in tonotopicity and in the balance of excitatory and inhibitory neuro transmission [10].

Noise-induced hearing loss (NIHL) is a public health problem happening during daily life, which is primarily disregarded, but may lead to the intense morbidity as it is developed. Noise-induced hearing loss (NIHL) is a complex disorder that may possibly be affected by environmental and genetic factors [11]. NIHL is a slowly progressive, sensorineural hearing deficit, which is typically occur at higher frequencies (3–6 kHz) as a result of the chronic exposure to excessive sound and it is one of the most usual forms of hearing loss in the United States existing in nearly one in four adults [12]. Various levels of hearing the loss in different people may be created by the similar intensity of noise with the same duration [11].

The National Institute of Occupational Safety and Health (NIOSH), USA, still classifies the noise-induced hearing loss among the top ten work-related problems, which involves at least 11 million workers in the US. It was reported in the recent studies that despite the hearing conservation programs, employees continue to develop NIHL [13]. Occupational noise-induced hearing loss (ONIHL) is still common: high-frequency hearing impairment was found to be significantly associated with self-reported “very loud” noise exposure at work in a 2011 to 2012 audiometric survey of the US adult population, (odds ratio = 2.0), even after regulating for age, gender, non-occupational noise, and other explanatory variables [14]. Air conduction (AC) and bone conduction (BC) are traditionally described as the two major paths of sound transmission to the inner ear. However, lately, studies on humans and experimental animals proved that hearing can also be caused by the response to soft-tissue stimulation [15].

Data mining (DM) is an interdisciplinary subfield of computer science. It is the computational procedure of discovering patterns in large data sets, which include the methods at the intersection of artificial intelligence, statistics, machine learning, and database systems. To extract information from a data set and convert it into an understandable structure for further use are the general objectives of the DM process. Such information may involve data classification and prediction of outcomes after an intervention, or it may investigate the association, group, or detection of variable deviation [16]. Our choice is motivated in two-fold mode. First, it is clear that data mining is something further than blindly utilizing algorithms to data hoping to discover something useful. For successful application of data mining, the domain knowledge and a clearly articulated objective or area of interest are required [17]. Additionally, the application of data mining to new domains often increases the interesting issues not controlled by the current methods well, and therefore offers opportunities for useful algorithmic development and extensions [18].

Rule sets are preferred in many applications, because of simplicity and easiness for understanding compared to the decision trees. C5 can create classifiers demonstrated either as decision trees or as rule sets. C5 algorithm follows the rules of the algorithm of C4.5, as well as the rules of the ID3 algorithm. C5 algorithm involves many features, as the large decision tree can be considered as a set of rules easy to understand, providing the knowledge on noise and missing data. In classification technique, problem of over fitting and error pruning are solved and the C5 classifier can determine the relevant and irrelevant attributes in classification. The algorithm C5 indicates that how the rule sets are created with improved features while generating fewer rules, therefore, compare to other classifiers, memory usage is low [19].

Because hearing loss is one of the most common noise exposure impairments, it results in high costs for workers in the industry and exposed individuals. Considering that, fewer studies exist in the world conducted on weighting and prioritizing factors affecting the workers hearing loss based on the audiometry data using the C5 algorithm, this study was designed to examine the following objectives:

- Determining the workers’ equivalent sound level.
- Determining the hearing loss of both ears.
- Determining the weight of the factors affecting hearing loss based on the C5 algorithm.
- Determining the error & accuracy rate of the C5 algorithm.

## Method

### Study site and sampling methods

This study was conducted in mining industry in southeastern Iran, for which according to the individual’s equivalent sound level and previous studies, as well as the type of algorithm used in hearing loss modeling, totally three groups (one control group and two case group) was considered. For each group, 50 people were assigned (according to the equivalent level of exposure to different sounds in every three groups). Thus, the total number of subjects under study was 150 [20].

### Designing the study

This is a cross-sectional, descriptive-analytic prospective study. The general stages in orders are as the following: 1) choosing the predictive factors; 2) performing the audiometry and calculating the permanent hearing loss of each ear and then overall hearing loss; 3) classifying the types of hearing loss (It should be noted that the audiometric data are divided into two parts, teaching and evaluation. In teaching part 40 people were selected from each group and so total were 120 persons, and in the evaluation part, 10 persons were selected from each group that subsequently amounted to 30 persons); 4) determining the weight of factors effective on the hearing loss based on the C5 algorithm; and 5) determining the error and accuracy rate [21]. Four variables including age, work experience, the equivalent sound level and frequency were considered for each person [21,22]. All participants were adults and were divided three age groups; the first age range was between 20 and 35 years, the second range was between 35 and 50 years, and the third age range was above 50 years [21]. In terms of the work experience, they were divided three ranges with a work experience of fewer than 10 years, between 10 to 20 years and over 20 years [20]. Measuring the equivalent sound level based on ISO 9612 was carried out using a dosimetry method and with a TES-1345 device made in Taiwan. Before using this device, a calibrator of 110/2 CEL made in England was utilized to calibrate the machine [23]. Furthermore, in this study, the frequencies of 500, 1000, 2000, and 4000 Hz were also considered as the effective factors (predictors) [24].

### Audiometry

Using Clinical Audiometer CA 120 made in Denmark, pure-tone hearing thresholds at 250, 500, 1000, 2000, 4000, 6000 and 8000 Hz were recorded [25].

### Calculating permanent hearing loss

At first, the permanent hearing loss of the left and right ear was calculated separately, for this aim, the hearing loss in each 4 important frequencies of 500, 1000, 2000, and 4000 was entered in the related equation after the fraction of age, and the rate of permanent hearing loss caused by sound was calculated (Equation 1). Subsequently, Equation 2 was used to calculate the total permanent hearing loss [23].

**Equation 1:**

NIHL = \frac{{\left({T{L_{500Hz}}} \right) + \left({T{L_{1000Hz}}} \right) + \left({T{L_{2000Hz}}} \right) + \left({T{L_{4000Hz}}} \right)}}{4}

**TL:** Hearing loss at the considered frequency per ear (dB)

**NIHL:** Permanent hearing loss caused by sound (dB)

**Equation 2:**

NIH{L_t} = \frac{{\left({NIH{L_b} \times 5} \right) + \left({NIH{L_p}} \right)}}{6}

**NIHL_t_:** Total permanent loss in both ears (dB)

**NIHL_b_:** permanent loss of the stronger ear (dB)

**NIHL_p_:** permanent loss of the poor ear (dB)

Hearing loss classification based on the WHO categorization is such that hearing in the range of 0–25 dBA was classified as normal hearing, 26–40 dBA as a mild loss, 41–60 dB as average loss, 61–80 dBA as severe loss and over 80 dBA was classified as deep loss [26].

### Determining the weight of the factors affecting hearing loss based on the C5 method and evaluating the accuracy of its models

**Figure 1:**
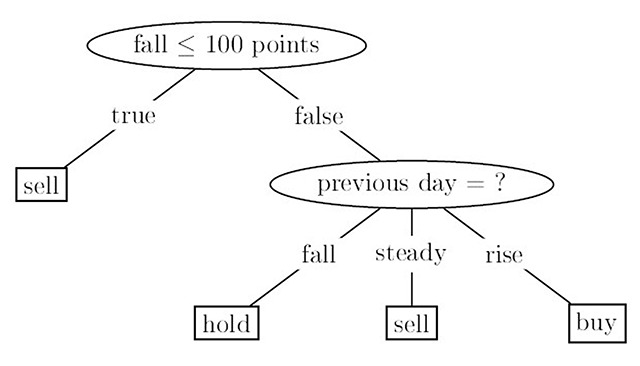
Decision tree algorithm Structure.

In this method tree, structure was used to create the classification models. A dataset is divided in this method into smaller subsets. Leaf node provides a decision. The decision trees classify the cases based on feature values of instances. Each node indicates a feature in an instance in a decision tree, which is to be classified, and each branch provides a value. Based on the feature values Classification of Instances starts from the root node and it is sorted. Decision trees can control categorical and numerical data [19] (Figure 1).

The objective is to discover classification rules determining the class label of any object (Y) from the values of its attributes (X). Each internal node (non-leaf node) provides a test condition on an attribute, an outcome of the test is obtained from each branch, and a class label is represented by each leaf node (or terminal node). Let a node N provides the tuples of partition D. The attribute with the highest information gain is selected as the splitting attribute for the node N. Through this attribute the information needed to classify the tuples in resulting partitions is minimized and the least randomness in these partitions is reflected. The expected information needed to classify a tuple in D is given as the following [27]:

**Equation 3:**

Info(D) = - \sum\nolimits_{i = 1}^m {{P_i}\log_{2}({P_i})}

Where *P_i_* represents the probability that an arbitrary tuple in D is associated with class *C_i_* and is estimated by |*C_i, D_*|/|*D*|. Info D is the average amount of information required to determine the class label of a tuple in D. Info (D) is also recognized as the entropy of D. Suppose that we to attempt to partition the tuples in D on an attribute A having υ distinct values {*a_1_, a_2_,…,a_υ_*}. The obtained partitions are associated to the branches of the node N. Info_A_ (D) is the expected information needed to categorize a tuple from D based on the partitioning by A.

**Equation 4:**

Inf{o_A}(D) = \sum\nolimits_{j = 1}^\upsilon {\frac{{\left| {{D_j}} \right|}}{{\left| D \right|}}} \times Info({D_j})

The term {\textstyle{{\left| {{D_j}} \right|} \over {\left| D \right|}}} acts as the weight of the *j* th partition. The information gain is explained as the difference between the original information necessity (i.e. based on just the proportion of classes) and the new necessity (i.e. obtained after partitioning on A).

**Equation 5:**

Gain({\rm A}) = Info(D) - Inf{o_A}(D)

The attribute A with the highest information gain, Gain (A), is selected as the splitting attribute at node N [27].

In classification algorithms that are used to categorize discrete-type output factors, the evaluation criteria such as accuracy, confusion matrix, sensitivity, and attribute are used. In this study, two criteria of accuracy and confusion matrix were used. The confusion matrix is a square matrix with the dimensions equal to the number of output factors classes. In this matrix, the main diameter represents the percentages that were correctly predicted. According to Equation 6, the accuracy of the model is actually the ratio of the correct predicted cases to the total cases [28].

**Equation 6:**

\boldsymbol{Accuracy} = \frac{\bf{True\ Postive\ cases} + \boldsymbol{True\ Negative\ cases}} {\bf{All\ cases}}

### Ethical considerations

The Ethics Committee of Kerman University of Medical Sciences approved the present work ethically (ID: IR.KMU.REC.1396.2458). All participants signed a consent form.

### Processing and analyzing the data

Using Statistical Package for Social Sciences V.18 (SPSS) (SPSS, Inc., Chicago, Illinois, USA), the collected data were analyzed. Mean, standard deviation and correlation coefficient were analyzed using linear regression statistical and paired t-test tests. Moreover, modeling the hearing loss changes was performed using IBM SPSS Modeler 18.0.

## Results

### Demographic information

The demographic data of participants are showed in Table 1.

**Table 1:** Demographic information of the study sample (n = 150).

	Variables	Mean	SD*

The first group (n = 50) (SPL <70 dBA)	Age	37.66	9.91
	Work Experience	9.1	4.9
The second group (n = 50) (SPL 70–80 dBA)	Age	35.56	11.45
	Work Experience	8.48	5.38
The Third group (n = 50) (SPL >85 dBA)	Age	41.76	10.93
	Work Experience	11.34	5.32

* SD = Standard deviation.

### Measuring the equivalent sound level

The first group was exposed to an equivalent sound level of less than 70 dB. The second group was exposed to the equivalent sound level of 70 to 80 dB, and the third group was exposed to the equivalent sound level over 85 dB. The mean and standard deviation of the equivalent sound level for the first, second and third groups were 70 ± 3 dBA, 77.62 ± 4.43 dBA, and 89.7 ± 3.03 dBA, respectively.

### Results of hearing loss

The results of the workers’ hearing loss in both ears based on the severity of hearing loss are provided in Table 2. A paired t-test showed that there was no significant statistical difference between the mean values of hearing loss in the right and left ears in similar frequencies in the three groups (P > 0.05).

**Table 2:** Classification the Participants Hearing Loss (n = 150).

	Normal (0–25 dB)	Moderate (41–60 dBA)	Mild (26–40 dBA)	Severe (61–80 dBA)	Profound (80 dBA <)

**The first group (n = 50)** **(SPL <70 dBA)**	40 participants (80%)	10 participants (20%)	–	–	–
**The second group (n = 50)** **(SPL 70–80 dBA)**	37 participants (74%)	10 participants (20%)	3 participants (6%)	–	–
**The third group (n = 50)** **(SPL >85 dBA)**	29 participants (58%)	15 participants (30%)	4 participants (8%)	2 participants (4%)	–

### Modeling the hearing loss changes based on C5 algorithm

In this study, four different models of hearing loss were calculated. In the first model, the Audiometric data of the first group workers (SPL <70 dBA), in the second model the Audiometric data of the second group workers (SPL 70–80 dBA), in the third model, the audiometric data of the third group workers (SPL >85 dBA), and ultimately, in the fourth model the total audiometric data of workers in the three groups were modeled.

### Model 1: Modeling the hearing loss changes in the audiometric data for the workers in the first group (SPL <70 dBA)

**Figure 2:**
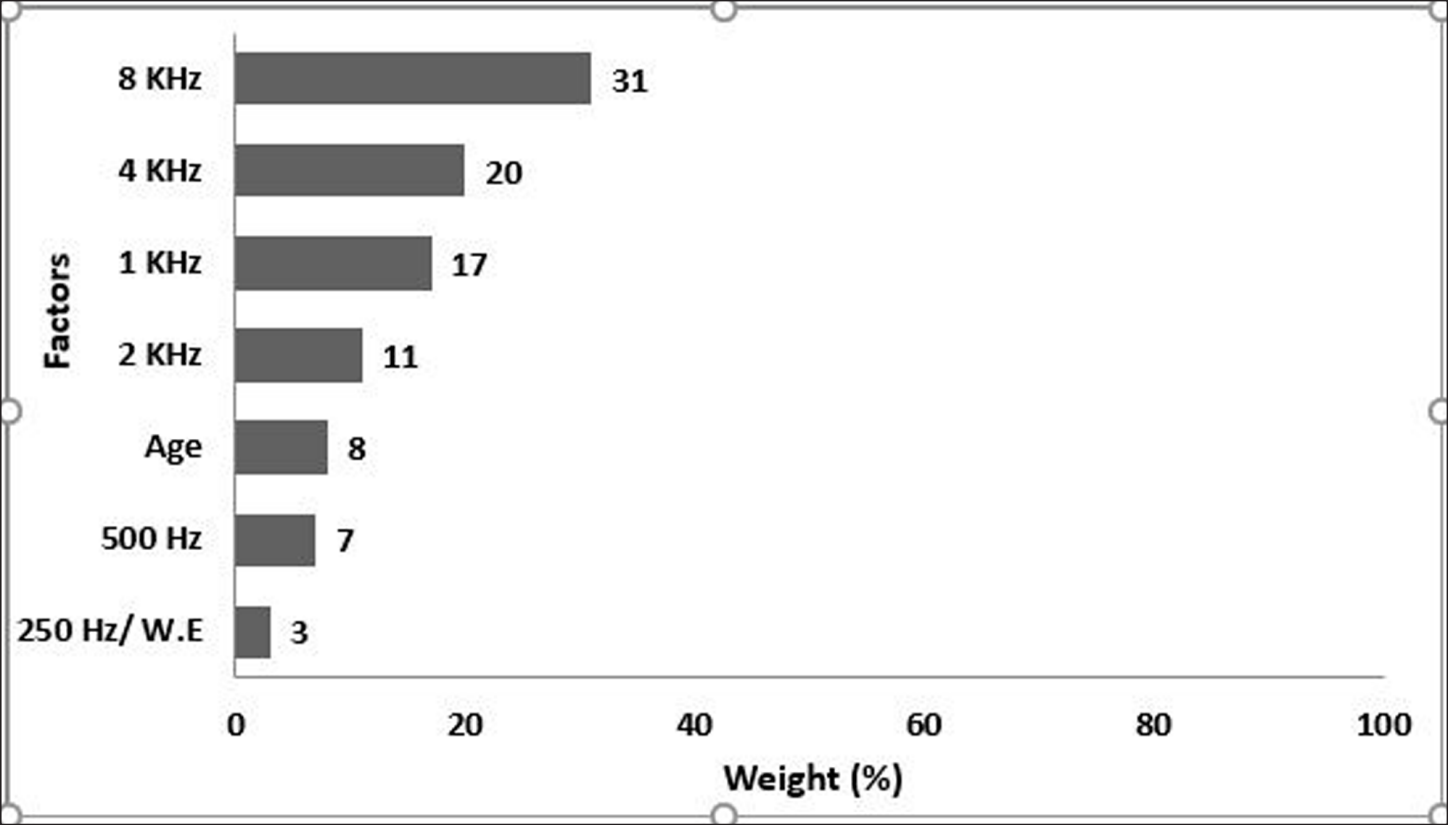
The first model, weight percent of hearing loss predictors in the first group (SP <70 dBA).

Inserting the results of the above factors for the first model (n = 10) in IBM SPSS Modeler 18.0, the results are obtained as shown in Figure 2. As it is observed, the 8KHz frequency with a weight of 31% has the highest effect, and 250Hz and work experience each with a weight of 3% have the lowest effects.

The model predicted correctly the severity of hearing loss for all people with the normal and mild hearing loss, and the accuracy of the C5 algorithm of 100% in this modeling.

### Second Model: Modeling the hearing loss changes for the workers audiometric data in the second group (SPL 70–80 dBA)

**Figure 3:**
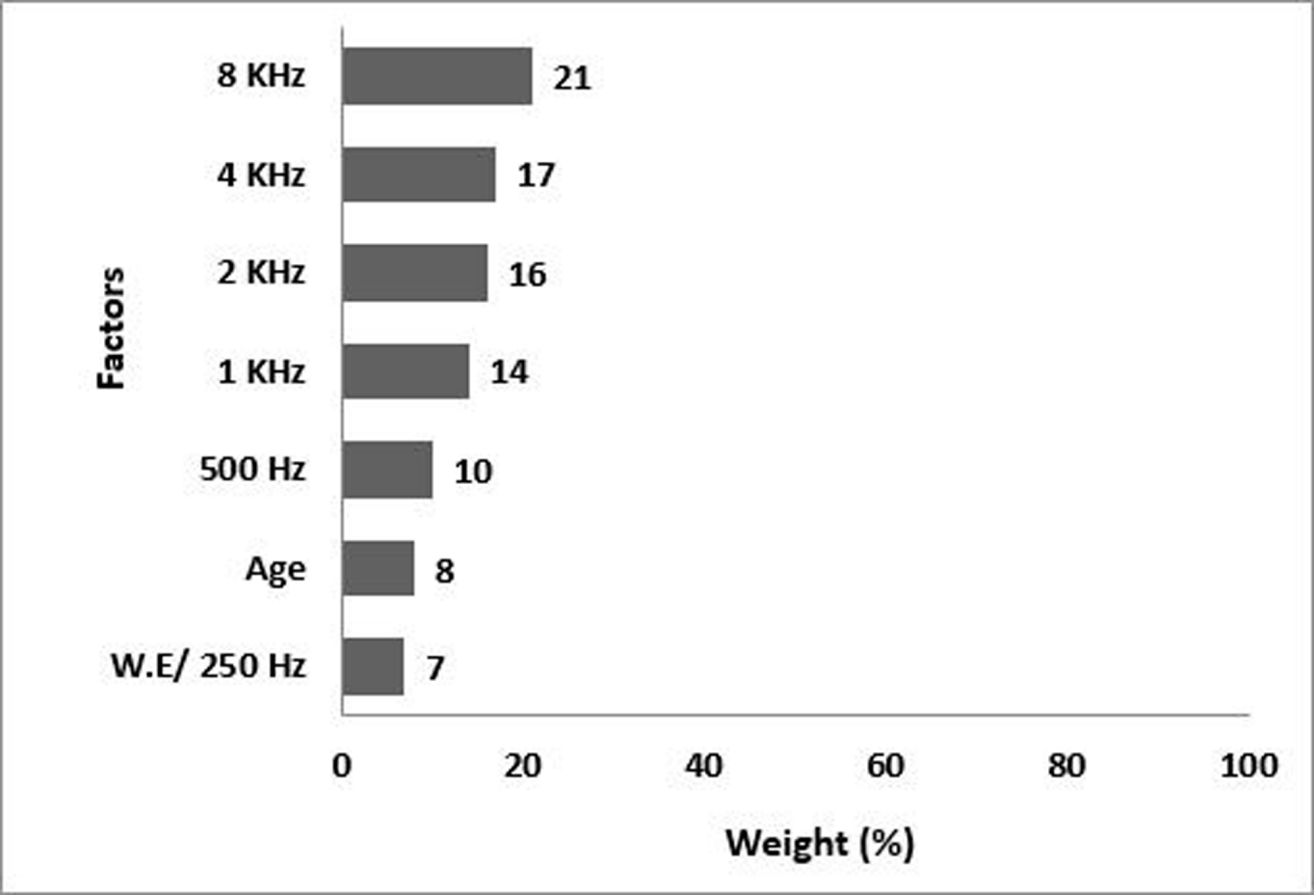
The second model, the weight percent of predictive hearing loss factors of the workers in the second group (SPL 70–80 dBA).

The results of modeling the hearing loss changes in the second model (n = 10) are shown in Figure 3. The 8KHz frequency with a weight of 21% has the highest effect, and the 4KHz frequency with a weight of 17% has the second highest effect. The 2KHz and 1KHz frequencies are the third and fourth leading factors in hearing loss, respectively. The frequency of 250Hz & work experience each with a weight of 7% have the lowest effects.

The model predicted correctly the severity of hearing loss of all people with normal, mild and moderate hearing loss, and the accuracy of the C5 algorithm was obtained 100% in modeling hearing loss changes.

### Third Model: Modeling the hearing loss changes for the workers audiometric data in the third group (SPL >85 dBA)

**Figure 4:**
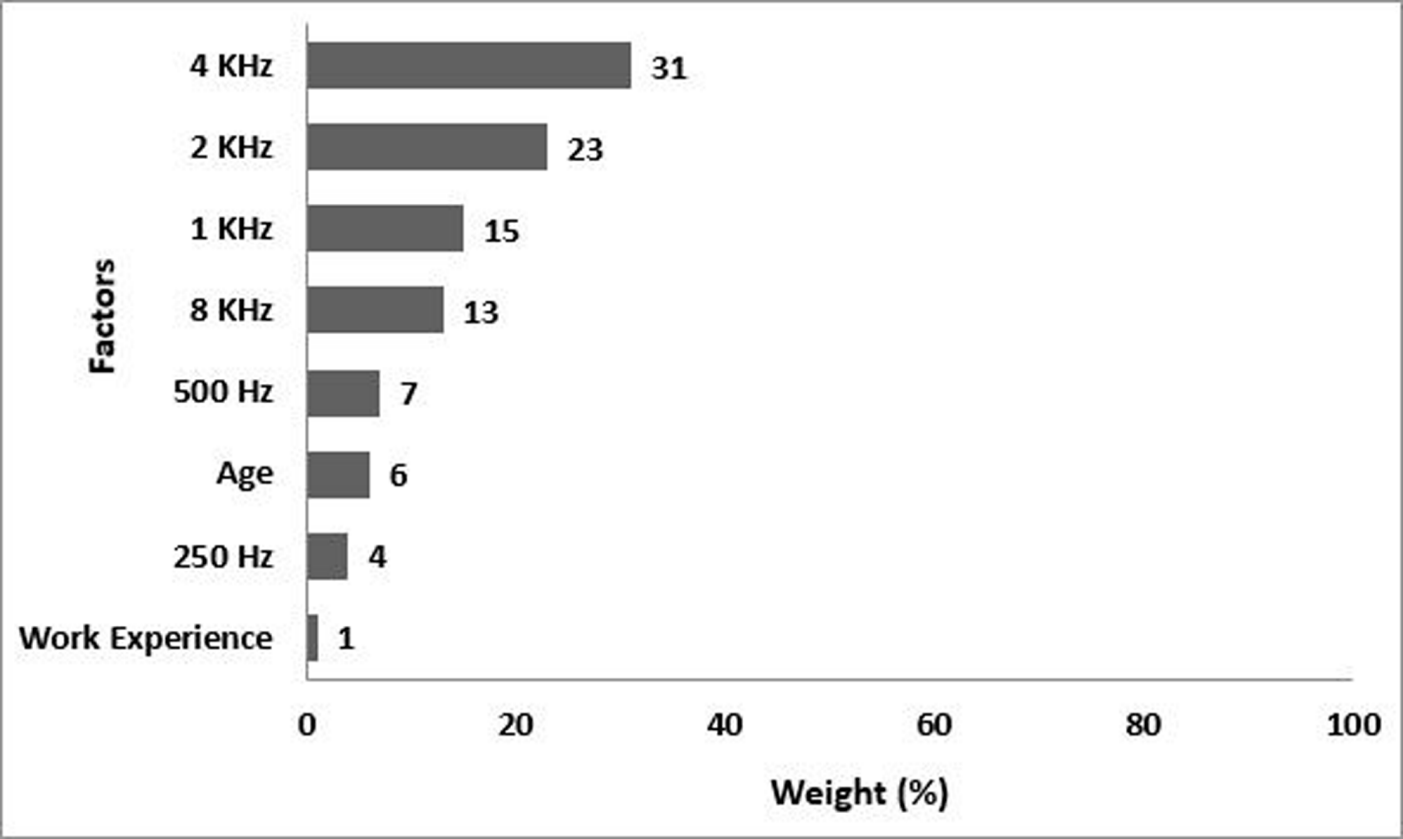
The third model, the weight percentage of the predictive hearing loss factors of workers in the third group (SPL >85 dBA).

The results of modeling the hearing loss changes in the third model (n = 10) are provided in Figure 4. The 4KHz frequency with the weight of 31% has the highest effect and the 2KHz frequency has the second highest effect (weighing 23%). Work experience with a weight of 1% has the lowest effect.

The model predicted accurately the severity of hearing loss of all people with moderate & severe hearing loss; 6.67% of the people with mild hearing loss were mistakenly predicted as the normal and 6.9% of those with normal hearing loss, were incorrectly predicted with mild hearing loss. The accuracy of the C5 algorithm was 94%.

### Fourth model: Modeling the hearing loss changes of the audiometric data in total workers’ groups

**Figure 5:**
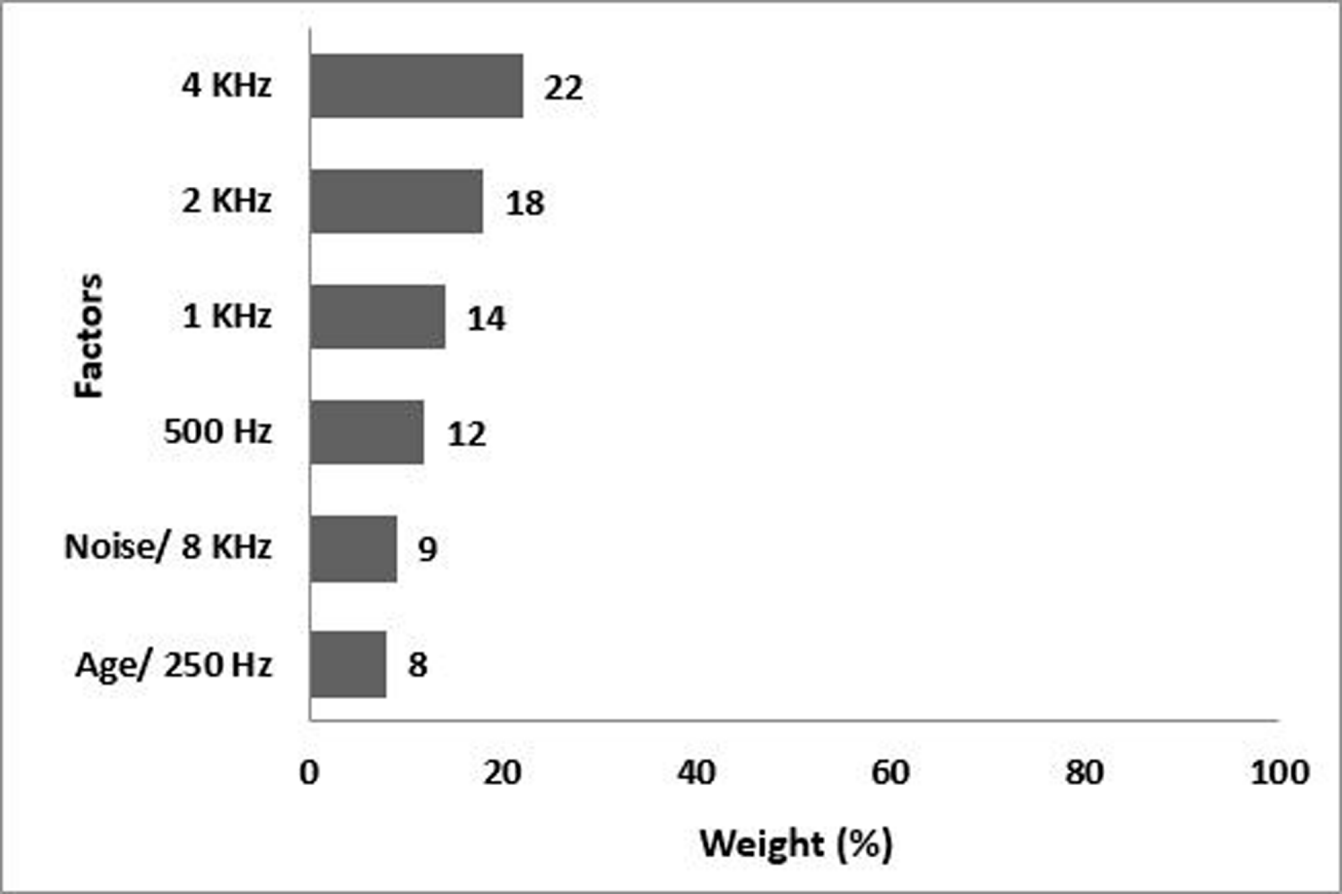
The fourth model, the weight percentage of the prediction factors of workers’ hearing loss in all three groups.

Inserting the results of the effective factors for the fourth model (n = 30) in the software, the results are obtained as shown in Figure 5. As it is seen, the frequency of 4KHz with a weight of 22% is the most effective, and frequencies of 2KHz, 1KHz, and 500Hz are second to fourth, respectively. The factor of age and 250Hz each with a weight of 8% have the least effects.

The model precisely predicted the severity of hearing loss for all people with mild, moderate, and severe. 0.95 percent of those with normal hearing loss, were incorrectly predicted as mild. The accuracy of model was 99.33% with an error rate of 0.67%.

## Discussion

This study aimed to use the C5 algorithm to determine the weight of factors affecting workers’ hearing loss based on the audiometric data. The mean exposure to equivalent sound level for the first, second and third groups was 70 ± 3 dBA, 77.62 ± 4.43 dBA, and 89.7 ± 3.03 dBA, respectively. There was a significant statistical difference between age and work experience with hearing loss in the first and second groups, so that in the first group between age and hearing loss, P = 0.008, R = 0.385 and in the second group, P = 0.008, R = 0.394. There was a statistically significant difference between work experience and hearing loss in the first group (P = 0.014, R = 0.362) and second group (P = 0.038, R = 0.32), too. However, in the third group, there was no significant statistical difference between age and working experience with hearing loss, as between the age and hearing loss, R = 0.189, P = 0.277, and between work experience and hearing loss, R = 0.28, P-value = 0.076. Based on the correlation and linear regression, there is a statistically significant difference between hearing loss and sound in all the subjects (n = 150), (P-value = 0.0001, R = 0.414).

In a study conducted by Aghilienejdad et al. to investigate the effect of work environment noise on workers’ hearing at small workshops in Tehran, in 2007, 109 people among the employees in the workshops with less than 50 workers were selected as a case group and the same number were selected as the control group considering all the aspects. In the case group, according to regression model coefficients and regression analysis results, the equivalent sound level was as the first factor, age, and work experience was considered as the next predictor of hearing loss. For the working people in the case group, the progression of hearing loss was associated with the increased work experience [29]. The results of the present study are closely related to the study of Aghilinejdad and increasing the work experience leads to the increased hearing loss. Dehghani et al. (2008) found similar results in a study to investigate the relationship between noise pollution and hearing loss among staff at Sarkhon gas refinery. first They identified the units above 85 dB, and then considered the staff of these units as the case group and the staff of other units as the control group. The results of statistical tests regarding the relationship between increasing age and work experience showed a positive and significant with hearing loss in both case and control groups [30]. In the present study, the increase in each of these two factors leads to the increased hearing loss.

According to C5 modeling, in the first model (SPL <70 dBA), the 8KHz frequency had the highest weight (31%), and the work experience and 250Hz each with a weight of 3% had the least effect (Figure 2). The accuracy of the C5 algorithm was 100% in the first model. In the second model (SPL 70- 80 dBA), the greatest effect on the hearing loss of people is associated to a frequency of 8KHz with a weight of 21%. the frequency of 250Hz and the working experience each with the weight of 7% had the minimum effect (Figure 3). In the second model, the accuracy of the C5 algorithm was 100%, too. In the third model (SPL >85 dBA), the 4KHz frequency with a weight of 31% had the greatest effect on hearing loss and the work experience with 1% weight had the lowest effect in this group (Figure 4), and the accuracy of the algorithm was 94%. In the fourth model, the frequency of 4KHz with a weight of 22% had the greatest effect on hearing loss, and the frequency of 250Hz and age had the smallest effect (8%) in this group (Figure 5). In this model, the accuracy of the algorithm was 99.33% with an error rate of 0.67%.

Exarchos et al. in 2016, analyzed the factors “epidemiologic, medical records, special disease records, laboratory findings, and clinical findings” of 985 patients from England, Belgium, Germany and Greece, they investigated the mining balance disorders’ data for developing the diagnostic decision support system through data mining technique. Patients suffered from imbalance disorders. Their goal was to create a diagnostic system for general practitioners and specialists to diagnose equilibrium disorders. The accuracy ranges from 59.3 to 89.8% for GPs and from 74.3 to 92.1% for experts [31]. The accuracy of the Exarchos’s study is relatively low inconsistent to our study. In the present study, the accuracy of the four decision tree models is high and close to 100%. Seixas et al. studied a Bayesian network decision model to support the diagnosis of dementia, Alzheimer’s disease, and mild cognitive impairment. They sought to provide a model using specialized information and data collected to diagnose demental disease, Alzheimer’s disease, and mild cognitive impairment. The bayesian network had the best result for mild cognitive impairment (97%) [32]. The accuracy obtained in this study is very high, similar to those of the present study.

Moreover, Miettinen et al. in 2008 in their study “classifying the otoneurological cases based on the Bayesian Probabilistic Models”, aimed to indicate the effectiveness of the Bayesian technique in classifying the otoneurological diseases and examining the attribute reliances. They considered a 38-variable set of data for classification. The accuracy of the Bayesian method was 97%, which was very high compared to other methods, such as the neural network [33]. The result of the present study was very high, as the study of Miettinen. Nawi et al. in 2011, predicted NIHL using the Gradient Descent through adaptive momentum (GDAM) algorithm and considering the factors of age, work experience, and occupational exposure as the main factors involved in hearing loss. They found the accuracy of predicting hearing loss for left ear 99.37% and for right ear 99.01%,22 which their acuuracy is close to our study accuracy, so that for the first two models the accuracy was 100%, for the fourth model was close to 100%, and for the third model, it was 94%. In the study of Acir et al. in 2005, on automatic cataloguing of auditory brainstem responses applying SVM-based feature selection algorithm for threshold detection, which was conformed using SVM algorithm on the findings, the obtained accuracy of the model was 96.2% [34]. The accuracy of the model in this study is also high consistent with the present work.

This study is innovative in terms of some aspects including the weighting of different factors such as sound pressure levels, different frequencies (250Hz, 500Hz, 1KHz, 2KHz, 4KHz, 8KHz), age and work experience. This is despite the fact that most studies for modeling the hearing loss changes in the world report only the error & accuracy rate and did not mention the weight and effect of each of these factors. Among the constraints of this study are the problems with the industry and not cooperating of some people in conducting audiometric tests.

## Conclusion

This article aimed to investigate the hearing loss and weight the factors affecting it by using C5 algorithm. According to the results in the first model, with the predictive factors (age, work experience, equivalent sound level exposure and frequency), the frequency of 8KHz with the weigh of 31%, had the highest effect and the factors of work experience & frequency of 250Hz each with a weight of 3% had the lowest effect. The accuracy of the predicted hearing loss model was also 100%. In the second model, considering the predictive factors, the frequency of 8KHz with a weight of 21% had the highest and the work experience & frequency of 250Hz each with a weight of 7% had the lowest effect; the accuracy of the predicted model was 100%. In the third model, the frequency of 4KHz with the weigh of 31% had the highest effect and the work experience with a weight of 1% had the lowest effect and the accuracy of the predicted model was 94%. Ultimately, in the fourth model, the frequency of 4kHz with the weight of 22% had the highest effect and the frequency of 250Hz and the age each with a weight of 8% had the lowest effect and the accuracy of the predicted model was 99.33%. Therefore, considering the high accuracy of the predicted models in the groups, the C5 algorithm is recommended as an appropriate and powerful tool for predicting and modeling the hearing loss.

## References

[1] Zare S, Nassiri P, Monazzam MR, Pourbakht A, Azam K and Golmohammadi T. Evaluation of the effects of occupational noise exposure on serum aldosterone and potassium among industrial workers. Noise and Health. 2016; 18(80): 1 DOI: 10.4103/1463-1741.174358 26780955 PMC4918676

[2] Nassiri P, Zare S, Monazzam MR, Pourbakht A, Azam K and Golmohammadi T. Evaluation of the effects of various sound pressure levels on the level of serum aldosterone concentration in rats. Noise and Health. 2017; 19(89): 200 DOI: 10.4103/nah.NAH_64_16 28816207 PMC5594925

[3] Zare S, Hasheminejad N, Shirvan HE, Hasanvand D, Hemmatjo R and Ahmadi S. Assessing individual and environmental sound pressure level and sound mapping in Iranian safety shoes factory. Rom J Acoust Vib. 2018; 15(1): 20–5.

[4] Nassiri P, Zare S, Monazzam MR, Pourbakht A, Azam K and Golmohammadi T. Modeling signal-to-noise ratio of otoacoustic emissions in workers exposed to different industrial noise levels. Noise and Health. 2016; 18(85): 391 DOI: 10.4103/1463-1741.174358 27991472 PMC5227021

[5] Zamanian Z, Rostami R, Hasanzadeh J and Hashemi H. Investigation of the effect of occupational noise exposure on blood pressure and heart rate of steel industry workers. J Environ Public Health; 2013 DOI: 10.1155/2013/256060 PMC3679689 23781252

[6] Kazemi R, Motamedzade M, Golmohammadi R, Mokarami H, Hemmatjo R and Heidarimoghadam R. Field study of effects of night shifts on cognitive performance, salivary melatonin, and sleep. Saf Health Work. 2018; 9(2): 203–9. DOI: 10.1016/j.shaw.2017.07.007 29928535 PMC6005914

[7] Zare S, Hemmatjo R, Allahyari T, et al. Comparison of the effect of typical firefighting activities, live fire drills and rescue operations at height on firefighters’ physiological responses and cognitive function. Ergonomics. 2018; 61(10): 1334–44. DOI: 10.1080/00140139.2018.1484524 29862929

[8] Safari Variani A, Ahmadi S, Zare S and Ghorbanideh M. Water pump noise control using designed acoustic curtains in a residential building of Qazvin city. Iran Occup Health. 2018; 15(1): 126–35.

[9] Han MA, Back SA, Kim HL, Park SY, Yeo SW and Park SN. Therapeutic effect of dexamethasone for noise-induced hearing loss: Systemic versus intratympanic injection in mice. Otol Neurotol. 2015; 36(5): 755–62. DOI: 10.1097/MAO.0000000000000759 25894725

[10] Jenkins KA, Fodor C, Presacco A and Anderson S. Effects of amplification on neural phase locking, amplitude, and latency to a speech syllable. Ear Hear. 2018; 39(4): 810–24. DOI: 10.1097/AUD.0000000000000538 29287038 PMC6014864

[11] Sliwinska-Kowalska M and Pawelczyk M. Contribution of genetic factors to noise-induced hearing loss: A human studies review. Mutat Res Mutat Res. 2013; 752(1): 61–5. DOI: 10.1016/j.mrrev.2012.11.001 23207014

[12] Vaisbuch Y, Alyono JC, Kandathil C, Wu SH, Fitzgerald MB and Jackler RK. Occupational noise exposure and risk for noise-induced hearing loss due to temporal bone drilling. Otol Neurotol. 2018; 39(6): 693–9. DOI: 10.1097/MAO.0000000000001851 29889779

[13] Borchgrevink HM. Does health promotion work in relation to noise? Noise Health. 2003; 5(18): 25. 12631433

[14] Dobie RA. Cost-effective hearing conservation: Regulatory and research priorities. Ear Hear. 2018; 39(4): 621–30. DOI: 10.1097/AUD.0000000000000523 29251690

[15] Chordekar S, Perez R, Adelman C, Sohmer H and Kishon-Rabin L. Does hearing in response to soft-tissue stimulation involve skull vibrations? A within-subject comparison between skull vibration magnitudes and hearing thresholds. Hear Res. 2018; 364: 59–67. DOI: 10.1016/j.heares.2018.03.030 29678325

[16] Guerra-Jiménez G, De Miguel ÁR, González JCF, Barreiro SAB, Plasencia DP and Macías ÁR. Cochlear implant evaluation: Prognosis estimation by data mining system. J Int Adv Otol. 2016; 12(1): 1 DOI: 10.5152/iao.2016.510 27340975

[17] Montgomery A. Data mining—business hunching, not just data Crunching. In: Proc of the Second International Conference on the Application of Knowledge Discovery and Data Mining PADD 1998; 39–48.

[18] won Lee J and Giraud-Carrier C. Results on mining NHANES data: A case study in evidence-based medicine. Comput Biol Med. 2013; 43(5): 493–503. DOI: 10.1016/j.compbiomed.2013.02.018 23566395

[19] Pandya R and Pandya J. C5.0 algorithm to improved decision tree with feature selection and reduced error pruning. Int J Comput Appl. 2015; 117(16). DOI: 10.5120/20639-3318

[20] Majumder J and Sharma LK. Application of data mining techniques to audiometric data among professionals in India. J Sci Res Reports. 2014; 3(23): 2860–971. DOI: 10.9734/JSRR/2014/12700

[21] Ramos-Miguel A, Perez-Zaballos T, Perez D, Falconb JC and Ramosb A. Use of data mining to predict significant factors and benefits of bilateral cochlear implantation. Eur Arch Oto-Rhino-Laryngology. 2015; 272(11): 3157–62. DOI: 10.1007/s00405-014-3337-3 25323153

[22] Nawi NM, Rehman MZ and Ghazali MI. Noise-induced hearing loss prediction in Malaysian industrial workers using gradient descent with adaptive momentum algorithm. Int Rev Comput Softw. 2011; 6(5): 740–8.

[23] Golmohammadi R and Aliabadi M. Noise and vibration engineering Daneshju; 1999.

[24] Schlauch RS and Nelson P. Puretone evaluation. Handb Clin Audiol. 2009; 6: 30–49.

[25] Gubbels SP, Gartrell BC, Ploch JL and Hanson KD. Can routine office-based audiometry predict cochlear implant evaluation results? Laryngoscope. 2017; 127(1): 216–22. DOI: 10.1002/lary.26066 27797418

[26] World Health Organization (WHO). Report of the Informal Working Group on Prevention of Deafness and Hearing Impairment Programme Planning, Geneva 1991; 18–21.

[27] Han J, Pei J and Kamber M. Data mining: Concepts and techniques. Elsevier. 2011.

[28] Marcoulides GA. Discovering knowledge in data: An Introduction to data mining. Taylor & Francis; 2005 DOI: 10.1198/jasa.2005.s61

[29] Aghilinezhad MA, Ali MI, Mohammadi S and Falahi M. Assessment of the effect of occupational noise on workers hearing in small scale industries in Tehran; 2007.

[30] Dehghani M, Pourjabbari AA and Ravandi MR. Relationship between noise pollution and hearing loss among workers in Sarkhoon Gas Refinery. Bimon J Hormozgan Univ Med Sci. 2012; 16(3): 181–8.

[31] Exarchos TP, Rigas G, Bibas A, et al. Mining balance disorders’ data for the development of diagnostic decision support systems. Comput Biol Med. 2016; 77: 240–8. DOI: 10.1016/j.compbiomed.2016.08.016 27619194

[32] Seixas FL, Zadrozny B, Laks J, Conci A and Saade DCM. A Bayesian network decision model for supporting the diagnosis of dementia, Alzheimer’s disease and mild cognitive impairment. Comput Biol Med. 2014; 51: 140–58. DOI: 10.1016/j.compbiomed.2014.04.010 24946259

[33] Miettinen K and Juhola M. Classification of otoneurological cases according to Bayesian probabilistic models. J Med Syst. 2010; 34(2): 119–30. DOI: 10.1007/s10916-008-9223-z 20433050

[34] Acır N, Özdamar Ö, Güzeliş C. Automatic classification of auditory brainstem responses using SVM-based feature selection algorithm for threshold detection. Eng Appl Artif Intell. 2006; 19(2): 209–18. DOI: 10.1016/j.engappai.2005.08.004

